# Site-selective doping effect, phase separation, and structure evolution in 1:1:1 triple-cation B-site ordered perovskites Ca_4−*x*_Sr_*x*_GaNbO_8_†

**DOI:** 10.1039/c9ra09970k

**Published:** 2020-01-08

**Authors:** He Huang, Pengfei Jiang, Wenliang Gao, Rihong Cong, Tao Yang

**Affiliations:** College of Chemistry and Chemical Engineering, Chongqing University Chongqing 401331 P. R. China pengfeijiang@cqu.edu.cn taoyang@cqu.edu.cn; College of Physics, Chongqing University Chongqing 401331 P. R. China

## Abstract

Oxygen-deficient perovskites are a family of important materials that may exhibit oxide ionic conductivities. We attempted to introduce oxygen-vacancy disordering in perovskite Ca_4_GaNbO_8_ (Ca_4_-type) by substituting Ca^2+^ with larger Sr^2+^. Sr^2+^-to-Ca^2+^ substitution did not lead to oxygen-vacancy ordering–disordering transition but an interesting Ca_4_-to-Sr_4_ type structure transition. Rietveld refinements revealed that the two-type structures exhibit similar oxygen-vacancy ordering and identical 1:1:1 triple-cation B-site ordering. Close inspection of the two-type structures revealed the subtle structure difference lies in the orientations of GaO_4_ tetrahedra, which is the origin of the formation of the narrow two-phase region (0.3 ≤ *x* < 0.65) in Ca_4−*x*_Sr_*x*_GaNbO_8_. More importantly, the A- and B-site cavities with large differences in size for both structures resulted in a site-selective doping behaviour for Sr^2+^ in Ca_4−*x*_Sr_*x*_GaNbO_8_. These structural changes found in Ca_4−*x*_Sr_*x*_GaNbO_8_ will provide a broad route approaching new oxygen-deficient phases with oxide ionic conductivities.

## Abstract 2

Sr^2+^-to-Ca^2+^ substitution resulted in new 1:1:1 triple-cation B-site ordered perovskites, where the structure difference lies in the orientations of GaO_4_-tetrahedra.

## Graphical abstract

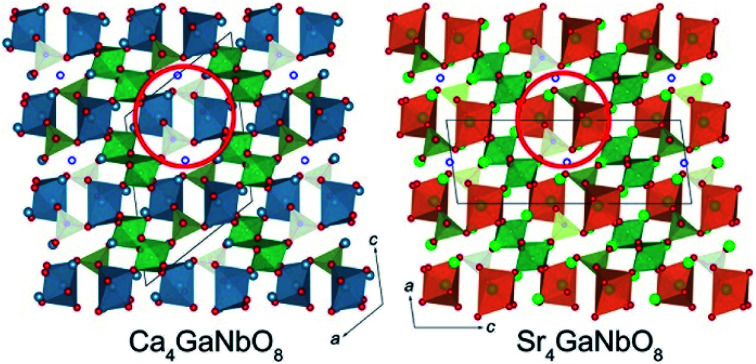

## Introduction

Perovskites with the general formula ABO_3_ can accommodate cations with a wide range of ionic radii and charge, thus giving rise to rich chemical compositions accompanied with diverse physical properties such as magnetism,^1,2^ superconductivity,^3,4^ dielectricity,^5,6^ ferroelectric.^7–9^ Such diversity in chemical compositions as well as physical properties for perovskites have motivated an intense and enduring interesting for chemists to design new materials and modify the physical properties of a specific phase by chemical substitution.

Among the various perovskites, 1:1 double-cation B-site ordering perovskites usually exhibit physical properties different from the disordered ones distinctively. For example, B-site ordered perovskites Sr_2_FeMoO_6_ and Sr_2_CrOsO_6_ exhibit colossal magnetoresistance and high temperature ferrimagnetism, respectively.^10,11^ In perovskites, B-site rock-salt ordering is commonly observed, because such an arrangement manner of B-site cations is benefit for keeping local charge neutrality and release of structure strain. In contrast, the layered ordered and columnar ordered perovskites are relatively rare, and only a few specific phases have been reported up to now.^12,13^

Owing to the structural flexibility, perovskite can accommodate various defects including A-site and oxygen vacancies, which can result in ionic diffusion at high temperature. A-site ionic diffusion was observed indeed in Li_3*x*_La_2/3−*x*_TiO_3_, in which the pre-existing A-site vacancies allow for easy Li^+^ diffusion.^14–16^ In contrast to A-site ionic conductivity, oxygen ionic conductivity or mixed ionic and electronic conductivities for oxygen deficient perovskites are much more widely investigated due to their potential applications in solid oxide fuel cells (SOFCs) as electrolyte or cathode. For example, Sr^2+^ and Mg^2+^ co-doped LaGaO_3_ (LSGM) is the one of the best oxygen ionic conducting electrolytes.^17–19^ Much efforts have been devoted to improve the oxygen ionic conductivities of various perovskites by increasing the number of oxygen vacancies. However, increasing the number of oxygen vacancies may result in completely ordering of oxygen vacancies, which in turn lead to a significant decrease of oxide ionic conductivity. For example, oxygen-vacancy ordered perovskite Ba_2_In_2_O_5_ exhibits poor ionic conductivity in low temperature range (<900 °C), though there exists a large number of oxygen vacancies in the structure.^20–22^ Oxygen-vacancy ordering/disordering in perovskite is closely related to the A-site cationic size (or tolerance factor, *t*). For example, with an increase of the Sr^2+^-content (increase of tolerance factor) in Ca_2−*x*_Sr_*x*_FeCoO_6−*δ*_ resulted in an oxygen-vacancy ordering to disordering transition.^23^ Therefore, the strategy of increasing the A-site cationic size of anion-ordered perovskites might be utilized to improve the oxide ionic conductivity.

Herein this contribution, our attention is turned to the newly discovered oxygen-deficient perovskite Ca_4_GaNbO_8_, where the complex 1:1:1 triple-cation B-site ordering is coupled with the oxygen-deficient ordering.^24^ We attempted to incorporate larger Sr^2+^ cations into Ca_4_GaNbO_8_ to bring in oxygen-vacancy disordering, so as to obtain new oxide ionic conductors. Substitution of Ca^2+^ with Sr^2+^ led to a Ca_4_-type to the Sr_4_-type structure transition as we expected. Unfortunately, Rietveld refinements manifested that the oxygen vacancies are also ordered in the Sr_4_-type structure, which was further confirmed by AC impedance spectroscopy measurements on selected compositions due to the absence of oxide ionic conductivity. The subtle structural differences between the Ca_4_-and Sr_4_-type structures lie in the orientations of GaO_4_ tetrahedra, which is the origin of the coexistence of two phases in a narrow region 0.3 ≤ *x* < 0.65. Moreover, a site-selective doping behaviour was observed for Sr^2+^ in Ca_4−*x*_Sr_*x*_GaNbO_8_, however the A-site cationic ordering is only in short-range for all compositions.

## Experimental section

### Synthesis

The polycrystalline samples Ca_4−*x*_Sr_*x*_GaNbO_8_ (*x* = 0–4) were prepared by high temperature solid-state reactions using calcium carbonate (CaCO_3_, 99.99%), strontium carbonate (SrCO_3_, 99.99%), gallium oxide (Ga_2_O_3_, 99.99%), niobium oxide (Nb_2_O_5_, 99.99%) as raw materials. The starting materials were preheated at 500 °C for 10 h to remove the absorbed moisture. Stoichiometric starting materials with a total weight of 1.2 g were weighted and mixed in an agate mortar by hands and then preheated at 950 °C to decompose the carbonates. After this calcination, the resultant white powders were further grinded and pressed into pellet with 13 mm diameter and then calcinated between 1200 °C and 1300 °C for 60 hours with intermediate grinding and pressing.

### X-ray diffraction

The phase purity of the samples was investigated by powder X-ray diffraction (PXRD). PXRD were performed on the PANalytical Empyrean diffractometer equipped with a PXIcel 1D detector (Cu Kα radiation). The operation voltage and current were 40 kV and 40 mA, respectively. The PXRD data for phase identification were collected with the setting of 30 s/0.0262°. High quality PXRD data, which were used for Rietveld refinements with the TOPAS-Academic V6 software,^25^ were collected with a setting of 200 s/0.0131°.

### AC impedance spectroscopy

AC impedance spectroscopy measurements for Ca_4−*x*_Sr_*x*_GaNbO_8_ (*x* = 0, 2, and 4) were carried out over temperature range from room temperature to 800 °C using a Solartron 1260A impedance phase analyzer with the frequency from 10^−1^ to 10^7^ Hz. Before the measurements, the pellets were coated with platinum pasts and then heated at 800 °C for 1 h to form electrodes.

### Raman spectroscopy

The Raman spectroscopy measurements were performed on the LabRAM HR Evolution spectrometer. The room temperature Raman spectra for Ca_4_GaNbO_8_ and Sr_4_GaNbO_8_ were measured over the range of 100–4000 cm^−1^ using the 325 nm line and with the light focus on the sample trough an optical lens. The spectral resolution was about 1–2 cm^−1^.

## Results and discussion

### Phase identification

**Fig. 1:**
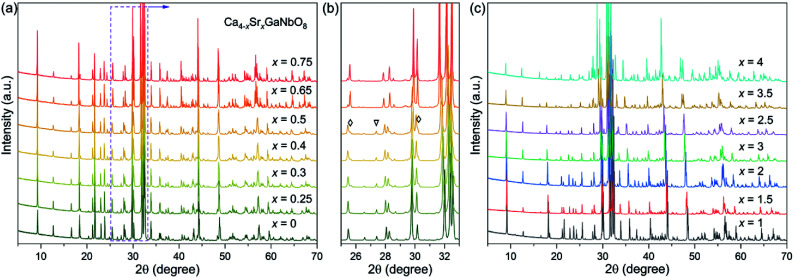
(a and c) Powder XRD patterns for Ca_4−*x*_Sr_*x*_GaNbO_8_ (*x* = 0–4). (b) The enlargement of 25–33° range for compositions *x* ≤ 0.65. The triangle represents the specific reflection ascribe to Ca_4_-type phase, the diamonds represent the reflections ascribe to Sr_4_-type phase.

PXRD data for Ca_4−*x*_Sr_*x*_GaNbO_8_ (*x* = 0–4) are elucidated in Fig. 1, where the diffraction peaks continuous evolves to lower angles upon increasing Sr^2+^-content. This observation indicates the cell volume expansion of Ca_4−*x*_Sr_*x*_GaNbO_8_ upon substituting Ca^2+^ with Sr^2+^, which is consistent with the larger cationic radii of Sr^2+^ (1.44 Å in 12-fold coordination) in comparison with Ca^2+^ (1.34 Å in 12-fold coordination).^26^

Previous study on Ca_4_GaNbO_8_ revealed that this compound adopts a triple-cation B-site ordered perovskite-type structure and crystallizes in *P*2_1_/*c* with lattice parameters *a* ≈ 11.18 Å, *b* ≈ 5.59 Å, *c* ≈ 14.07 Å, and *β* ≈ 121.55°.^24^ Our preliminary Le-bail fitting performed on Ca_4−*x*_Sr_*x*_GaNbO_8_ manifested that the XRD data for compositions with *x* < 0.65 could be indexed by this monoclinic cell. However, a group of weak reflections for compositions with *x* ≥ 0.65, which is not ascribe to impurity phases, could not be fitted with this monoclinic cell any more, indicating Sr^2+^-doping induced a change of lattice parameters. Indexing the XRD data of Ca_3_SrGaNbO_8_ yield a monoclinic cell with lattice parameters *a* ≈ *c*_1_/2, *b* ≈ *b*_1_, *c* ≈ √3*a*_1_, *β* ≈ 97°, where *a*_1_, *b*_1_, and *c*_1_ represent the cell dimension of Ca_4_GaNbO_8_ (denoted as Ca_4_-type phase). Similar unit cell was also observed for Sr_4_AlNbO_8_ (*P*2_1_/*c*), which also adopt a triple-cation B-site ordered perovskite-type structure with anionic vacancy ordering, suggesting Ca_4−*x*_Sr_*x*_GaNbO_8_ (*x* ≥ 0.65) (denoted as Sr_4_-type phase) is isostructural to Sr_4_AlNbO_8_.^27^

Preliminary Rietveld refinements performed on Ca_4−*x*_Sr_*x*_GaNbO_8_ (*x* = 0.5) using Ca_4_GaNbO_8_ as the initial structure model resulted in unreasonable structural parameters, *i.e.* too short interatomic distances. Close inspection of the XRD data revealed that some reflections from the Sr_4_-type phase were observed for compositions *x* = 0.3, 0.4 and 0.5. To clarify this clearly, the diffraction components from Cu Kα_2_ were striped for comparison. As shown Fig. 1b, small shoulders, corresponding to contribution of the Sr_4_-type phases, of some characteristic reflections were visually observed. Moreover, a representative Le-bail fitting pattern for *x* = 0.5 presented in Fig. S1† demonstrated that all the reflections could only be well reproduced by a two-phase model fitting. These results indicate that the compositions for *x* = 0.3, 0.4 and 0.5 comprise two phases. More importantly, single-phase for compositions *x* = 0.3, 0.4 and 0.5 was not attainable by neither calcination at elevate temperature nor prolongation of calcination time (Fig. S2†), suggesting the two phases are thermodynamically favorable. Therefore, the compositions for Ca_4−*x*_Sr_*x*_GaNbO_8_ (*x* = 0–4) can be divided into three regions: (i) a single Ca_4_-type phase region (*x* < 0.3), (ii) a narrow two-phase region contains both Ca_4_-type and Sr_4_-type phases (0.3 ≤ *x* < 0.65), and (iii) a single Sr_4_-type phase region (*x* ≥ 0.65). We should note that the change of the relative content for the two phases within the two-phase region was slight (Fig. 1b), which is distinctly differ from commonly observed two-phase regions for perovskites *e.g.* the two-phase region observed in Ba_3−*x*_Sr_*x*_ZnSb_2_O_9_ (0.3 ≤ *x* ≤ 1.0).^28^ Such a difference is attribute to the subtle distinction in crystal structures between Ca_4_-type and Sr_4_-type structures, which will be discussed later.

**Fig. 2:**
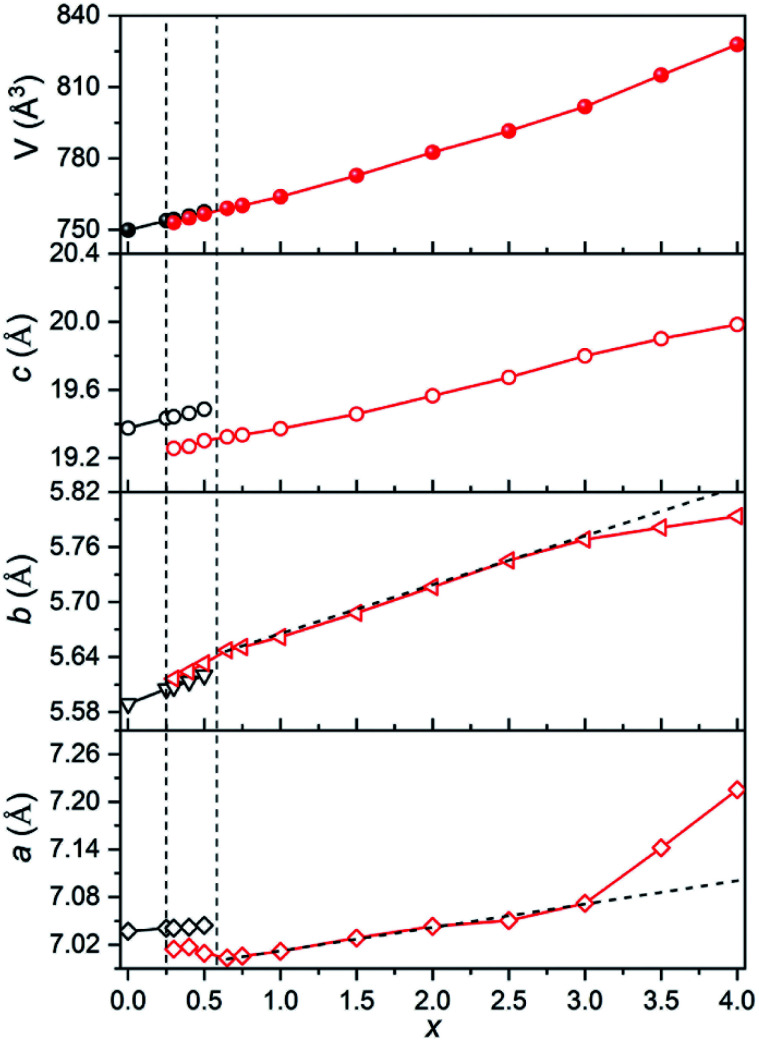
Plots of lattice parameters against Sr^2+^-content in Ca_4−*x*_Sr_*x*_GaNbO_8_.

The evolution of normalized lattice parameters against Sr^2+^-content in Ca_4−*x*_Sr_*x*_GaNbO_8_ is presented in Fig. 2, where the linear expansion of the cell volume for single-phase compositions is in good agreement with the PXRD patterns. Interestingly, the lattice parameters for both Ca_4_-type and Sr_4_-type phases also showed a linear increase within the two-phase region, which opposite to commonly observed constant lattice parameters for the two phases in the two-phase region. This uncommon phenomenon further corroborated that the co-existence of two phases in Ca_4−*x*_Sr_*x*_GaNbO_8_ is thermodynamically favorable.

### Rietveld refinements

**Fig. 3:**
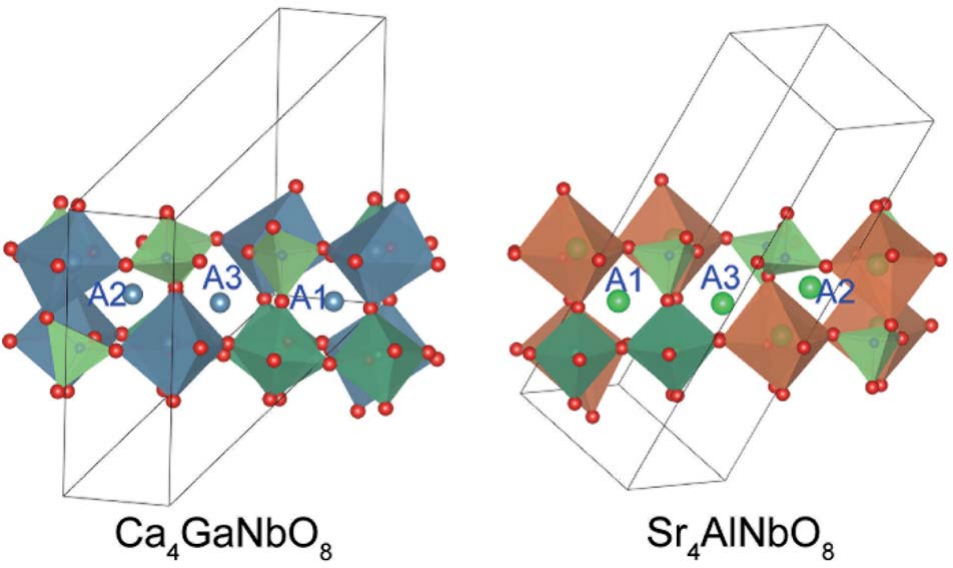
The surrounding environments for A-site cations in Ca_4_-type and Sr_4_-type structures.

As described above, both Ca_4_-type and Sr_4_-type phases adopt oxygen-deficient perovskite-type structure with 1:1:1 triple-cation B-site ordering. Owing to their low structure symmetry (*P*2_1_/*c*), both structures exhibit three distinctly different A-sites (denoted as A1, A2, and A3, respectively), which could be readily discerned through their surrounding environments. As shown in Fig. 3, A1 cation is surrounded by three B1O_6_ octahedra (B1 represents the B-site occupied by alkali earth cation), four NbO_6_ octahedra, and one GaO_4_ tetrahedron; A2 is surrounded by three B1O_6_ octahedra, one NbO_6_ octahedron, and four GaO_4_ tetrahedra; A3 is surrounded by two B1O_6_ octahedra, three NbO_6_ octahedra, and three GaO_4_ tetrahedra. Such difference in surrounding environments for A-site cations results in the A1-site is much larger than A2-and A3-site, suggesting Sr^2+^ is prone to occupy the A1-site at first.

**Fig. 4:**
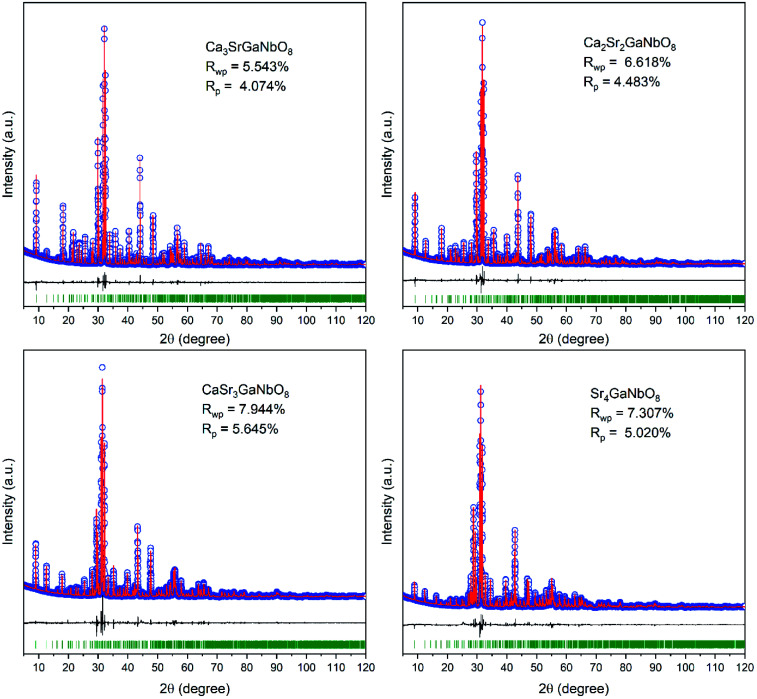
Rietveld refinement plots of XRD data for Ca_4−*x*_Sr_*x*_GaNbO_8_ (*x* = 0, 1, 2, 3, 4).

Careful Rietveld refinements against laboratory XRD data were further performed on selected compositions Ca_4−*x*_Sr_*x*_GaNbO_8_ (*x* = 0, 1, 1.5, 2, 2.5, 3, 3.5 and 4) to get an insight picture of the site occupancy preference for Sr^2+^. The refined structure parameters for Ca_4_GaNbO_8_ is in good agreement with that reported by Yang *et al.* using combined refinements against neutron and synchrotron data (Table S1†). For compositions with *x* ≥ 1.0, Sr_4_AlNbO_8_ was used as the starting structure model for Rietveld refinements. At first, an A-site ordered structure model was constructed for preliminary refinements, for instance, Sr^2+^ occupies A1-site exclusively and the remaining two A-sites were occupied by Ca^2+^ in Ca_3_SrGaNbO_8_. The refinement proceeded smoothly with this completely ordered model, resulting in reliable agreement factors (*R*_wp_ = 7.625%, *R*_p_ = 5.592%) and structural parameters. However, some peaks with large discrepancies between the observed and calculated were observed, suggesting the A-site cations are not completely ordering in Ca_3_SrGaNbO_8_. Consequently, the occupancy factor for Ca^2+^ and Sr^2+^ cations at B1-site and three A-sites were refined freely during the subsequent refinements. It turned out that A1-site was dominantly by Sr^2+^, and A2, A3, and B1 sites were mainly occupied by Ca^2+^. Despite the occupancies for Ca^2+^ and Sr^2+^ at four independent sites were refined freely, the refined composition Ca_2.92_Sr_1.08_GaNbO_8_ agrees well with the nominal formula Ca_3_SrGaNbO_8_. Moreover, the reliable factors were improved significantly to *R*_wp_ = 5.543% and *R*_p_ = 4.074%. These results manifest that the Ca^2+^ and Sr^2+^ are partially ordered in Ca_3_SrGaNbO_8_. Rietveld refinements performed on other compositions further demonstrated that all the compositions with mixed A-site cations were partially ordered. We should note that the inversion between A- and B-sites, which is commonly observed in spines,^29^ was not considered in Ca_4−*x*_Sr_*x*_GaNbO_8_ because of the large differences in cationic size and coordination environment preference between (Ca, Sr) and (Ga, Nb). The final crystallographic parameters and selected bond lengths for Ca_4−*x*_Sr_*x*_GaNbO_8_ are summarized in Table S1 and S2.† The Rietveld refinement patterns are presented in Fig. 4 and S3.†

### Structure evolution of Ca_4−*x*_Sr_*x*_GaNbO_8_

**Fig. 5:**
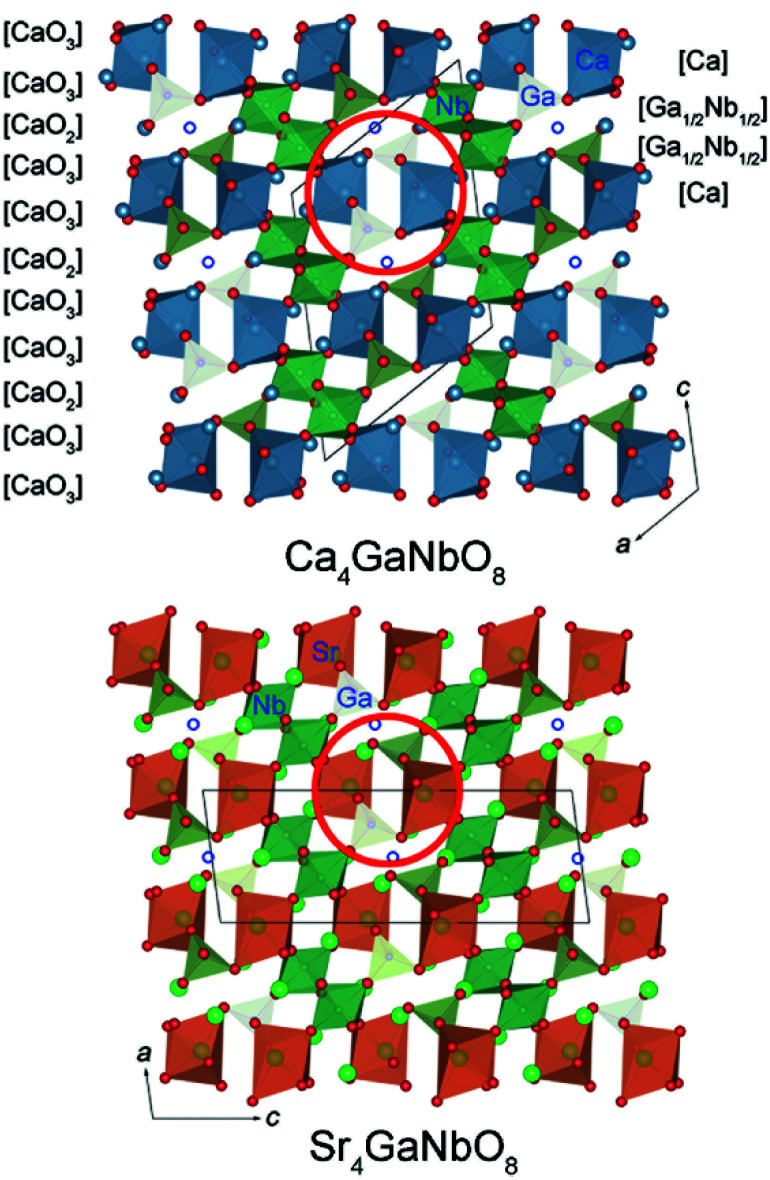
Comparison of crystal structures for Ca_4_GaNbO_8_ and Sr_4_GaNbO_8_. The open blue circles represent the oxygen-column defects.

The refined crystal structures for Ca_4_GaNbO_8_ and Sr_4_GaNbO_8_ are presented in Fig. 5, where both structures view along the same cubic-perovskite direction [110]_p_. Ca_4_GaNbO_8_ and Sr_4_GaNbO_8_ share the similar structure features with 1:2 layer-ordered hexagonal perovskites *i.e.* Ca_4_Nb_2_O_9_, however the structure for Ca_4_GaNbO_8_ and Sr_4_GaNbO_8_ are more complex because there is an another B-site cation (Ga^3+^) and ordered anionic deficiency.^30^ As shown in Fig. 5, the removal of oxygen within the AO_2_ layers creates tetrahedral cavities that could only accommodate smaller Ga^3+^ cations, which resulted in layered stacking sequence of (Ca/Sr)–(Ga_1/2_Nb_1/2_)–(Ga_1/2_Nb_1/2_)–(Ca/Sr) for B-site cations along the closed-packing direction [111]_p_. Owing to the ordered removal of every other oxygen-only O_2_ columns within the AO_2_ layers, Ga^3+^ and Nb^5+^ within (Ga_1/2_Nb_1/2_)–(Ga_1/2_Nb_1/2_) layers are column ordered along [110]_p_.

Given the same cationic ordering and oxygen deficient manner, the crystal structures for Ca_4_GaNbO_8_ and Sr_4_GaNbO_8_ seem identical at first glance. Close inspecting the structures revealed that the structural difference between Ca_4_GaNbO_8_ and Sr_4_GaNbO_8_ stems from the distinct orientations of GaO_4_ tetrahedra along-side of (Ca/Sr)O_6_ octahedra. As highlighted in Fig. 5, the GaO_4_ tetrahedra in Ca_4_GaNbO_8_ point to the same direction, however, the GaO_4_ tetrahedra in Sr_4_GaNbO_8_ point to opposite directions. Such a difference resulted in a doubled cell dimension along [111]_p_ for Ca_4_GaNbO_8_ in comparison with Sr_4_GaNbO_8_ (see Fig. 5). The structural transformation between two-type structures requires the rearrangement of the orientations of GaO_4_ tetrahedra along [110]_p_, which is much more difficult than collective octahedra-tilting observed universally in cubic-type perovskites. Consequently, a narrow two-phase region is observed in Ca_4−*x*_Sr_*x*_GaNbO_8_, and continuous structural transition is usually observed for simple cubic-type perovskites. We thus can speculate that this subtle structural difference in GaO_4_ orientations is the origin of the formation of the two-phase region in Ca_4−*x*_Sr_*x*_GaNbO_8_.

**Fig. 6:**
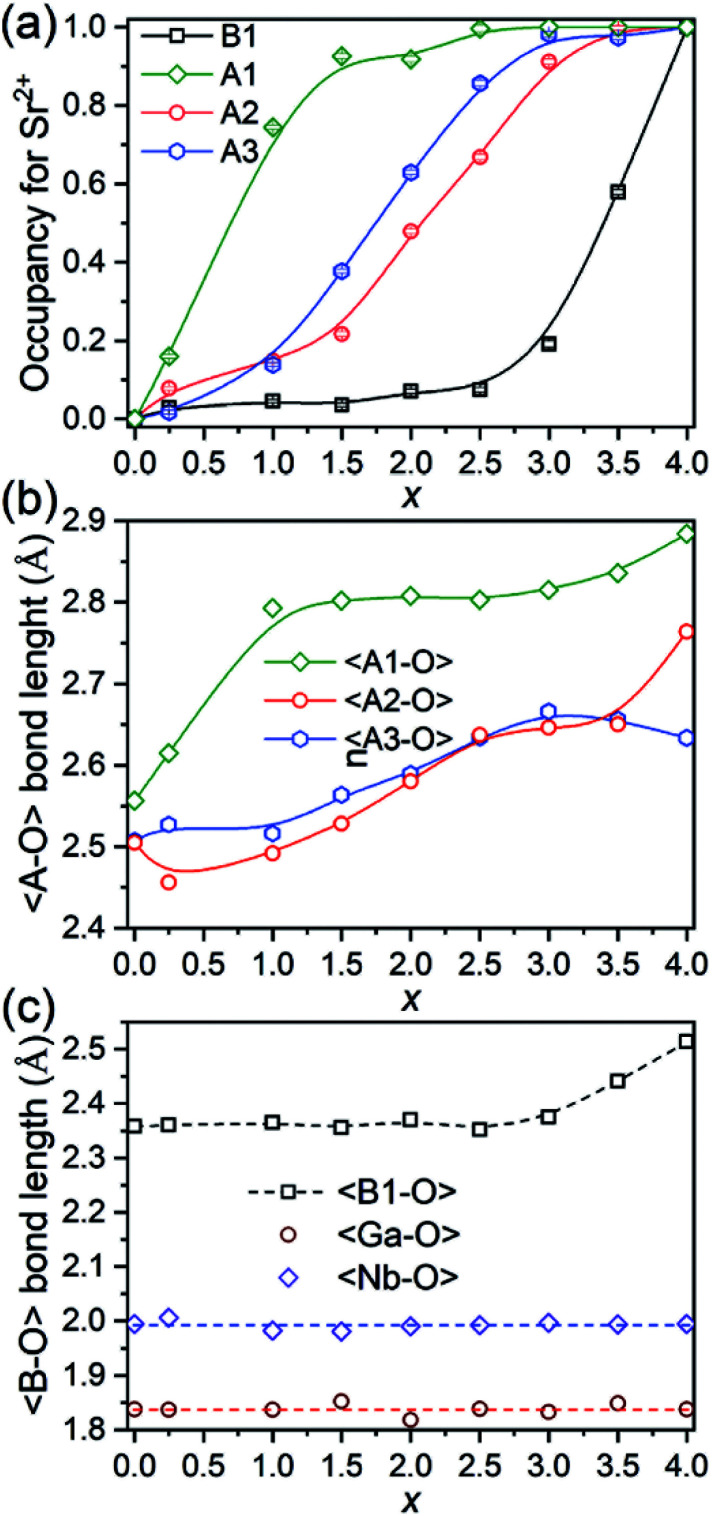
(a) Plots of occupancy factors for Sr^2+^, (b) average 〈A–O〉 bond lengths, and (c) average 〈B–O〉 bond length along with Sr^2+^-content in Ca_4−*x*_Sr_*x*_GaNbO_8_.

The evolution of occupancy factors for Sr^2+^ cations at both A and B sites in Ca_4−*x*_Sr_*x*_GaNbO_8_ is elucidated in Fig. 6a, where a site-selective doping behaviour is observed clearly. In detail, Sr^2+^ prefers to occupy the A1-site, which can be deduced from the sharp increase of occupancy factor to 0.926(6) for Sr^2+^ at A1-site when *x* ≤ 1.5, whereas the increase of occupancy factors at A2 and A3 sites are relatively slow. The occupancy factors for Sr^2+^ at A2 and A3 sites show a synchronized increase behaviour in the range of 1.5 ≤ *x* ≤ 3.0, where the Sr^2+^-occupancy at A1 site manifests a slight increase. Further incorporation of Sr^2+^ into Ca_4−*x*_Sr_*x*_GaNbO_8_ lead to a sharp increase of occupancy factor for Sr^2+^ at B1 site when *x* ≥ 3.0, whereas a slight increase is observed for *x* ≤ 3.0. Such a sharp increase of occupancy of Sr^2+^ at B1-site lead to a significant deviation of the lattice parameters, especially for *a*, from the Vegard's law (see Fig. 2). Given the layered structure nature of Ca_4−*x*_Sr_*x*_GaNbO_8_, when viewed along [110]_p_, the sharp increase of Sr^2+^-content in B1-site would unambiguously result in a sharp expansion along [111]_p_, namely *a*-axis of Sr_4_-type structure (see Fig. 5).

As described above, Sr^2+^ showed a site-selective doping behaviour due to the distinctly large differences in size for A1, A2/A3, and B1 sites. The evolutions of the average 〈A–O〉 and 〈B–O〉 bond lengths are elucidated in Fig. 6b and c, where the change trends for both 〈A–O〉 and 〈B1–O〉 bonds are in good agreement with that of occupancy factors. The 〈Ga–O〉 and 〈Nb–O〉 bond lengths are almost kept in constant at ∼1.85 Å and ∼2.0 Å, respectively, for all compositions (Fig. 6c). The Ga–O bond lengths in all compounds are in the range of 1.76–1.90 Å, which are comparable with four-coordinated Ga^3+^ in LaAGa_3_O_7_ (A = Ca^2+^, Sr^2+^, Ba^2+^).^31–33^ Detailed inspection of the Nb–O bonds revealed that the Nb^5+^ exhibits a distorted coordination environment with three Nb–O atomic distances shorter than 2.0 Å and the remaining three bond lengths longer than 2.0 Å (Table S2†), indicating Nb^5+^ displaced from the centre of the octahedral cavity due to the second-order Jahn–Teller (SOJT) effect.^34,35^ Though both Ga^3+^ and Nb^5+^ cations can adopt four- and six-fold coordinations, incorporation of Sr^2+^ into A- and B-sites did not lead to anti-site disordering between Ga- and Nb-sites for all compositions, which should unambiguous attribute to their large differences in charge and cationic size.

It is well known that Raman scattering is sensitive to local structural changes, including cationic ordering, structure symmetry change, and John–Teller distortion.^36^ To gain an insight of structural change induced by Sr^2+^-doping, Raman spectra for Ca_4_GaNbO_8_ and Sr_4_GaNbO_8_ were measured. Raman spectra of Ca_4_GaNbO_8_ and Sr_4_GaNbO_8_ show similar features (Fig. S4†), which is consistent with their similar crystal structures. The intensive peaks with wavelength numbers higher than 700 cm^−1^, especially for the strongest peaks with frequency near 800 cm^−1^, are characteristic features for the complex perovskites with B-site ordering.^30^ Moreover, the broad band in the middle frequency range of 530–610 cm^−1^ is assigned to be the stretching vibration of Nb–O bonds due to the displacement of Nb^5+^ from the centre of NbO_6_ octahedra.^37^ All these observations from the Raman spectra are coherent with the crystal structures for Ca_4_GaNbO_8_ and Sr_4_GaNbO_8_ obtained by Rietveld refinements.

### Transport properties

**Fig. 7:**
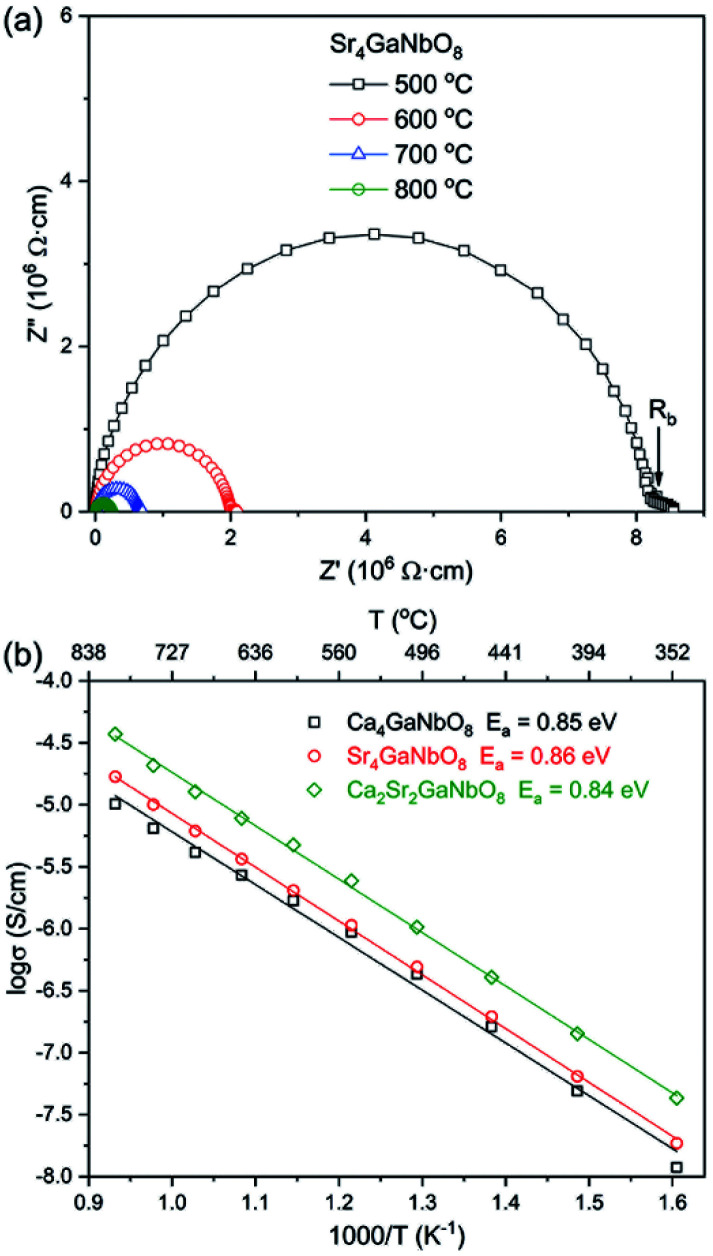
(a) Typical ac impedance spectra for Sr_4_GaNbO_8_ at different temperatures. (b) Arrhenius plots of bulk conductivities for Ca_4−*x*_Sr_*x*_GaNbO_8_ (*x* = 0, 2, and 4).

Typical AC impedance spectra at different temperatures for Ca_4−*x*_Sr_*x*_GaNbO_8_ (*x* = 0, 2, and 4) are presented in Fig. S5† and 7a, where the well-resolved semicircle in the high frequency rang can be modelled with parallel resistance (*R*) and capacity (*C*) element. This large semicircle is associated with a capacity close to 1 × 10^−12^ F cm^−1^, implying the contribution of bulk response. The absence of inclined-like spike in low frequency range indicates the absence of oxide ionic conductivity. This observation is consistent with the structural features that there are no terminal oxygens, which is capable of immigration at high temperature, boned to only one B-site cation in both Ca_4_-and Sr_4_-type structures. Moreover, the oxygen-vacancy ordering is coupled with the B-site cation ordering, which could not be disrupted upon warming. Therefore, the absence of oxide ionic conductivity for Ca_4−*x*_Sr_*x*_GaNbO_8_ is understandable. The plots of conductivities for Ca_4−*x*_Sr_*x*_GaNbO_8_ (*x* = 0, 2, and 4) against temperature is elucidated in Fig. 7b. All the compounds exhibit typical semi-conductive behaviour with comparable bulk conductivities (10^−8^–10^−4^ S cm^−1^) in the measured temperature range. Fitting the *σ*–*T* curves with Arrhenius equation gives active energy of ∼ 0.85 eV for all compounds, which in turn corroborated that the B-site cationic ordering is not disrupted by Sr^2+^-to-Ca^2+^ replacement.

## Conclusions

Substitution of Ca^2+^ in Ca_4−*x*_Sr_*x*_GaNbO_8_ with larger Sr^2+^ did not lead to oxygen-vacancy and B-site cation ordering–disordering transition but a Ca_4_-type (*x* < 0.3) to Sr_4_-type (*x* ≥ 0.65) structure transition across a narrow two-phase region (0.3 ≤ *x* < 0.65). Rietveld refinements revealed that two-type structures possess similar anionic ordering and identical B-site ordering. The structural difference only lies in different orientations of GaO_4_ tetrahedra, which is responsible for the formation of the narrow two-phase region. In the process of changing the Ca_4_-phase to Sr_4_-phase, the Sr^2+^ cations were mainly incorporated into A1-site for *x* < 1.5, and then doped into A2/A3-site for *x* < 3, and finally doped into B1-site (*x* ≥ 3). Such site-selective doping behaviour observed in Ca_4−*x*_Sr_*x*_GaNbO_8_ was driven by the distinctly large difference in size for A1, A2/A3, and B1 sites. Incorporation of Sr^2+^ cations into both A and B sites had no influence on the arrangement of Ga^3+^/Nb^5+^ cations and oxygen defects, thus no significant change in electronic properties of Ca_4−*x*_Sr_*x*_GaNbO_8_ was detected.

## Conflicts of interest

There are no conflicts to declare.

## Supplementary Material

RA-010-C9RA09970K-s001
