# Supplementary material for: Site-selective doping effect, phase separation, and structure evolution in 1:1:1 triple-cation B-site ordered perovskites Ca4−xSrxGaNbO8
Source: RSC Adv. 2020 Jan 8;10(4):1883–9. doi: 10.1039/c9ra09970k (PMC9047366; doi:10.1039/c9ra09970k)
Supplement: RA-010-C9RA09970K-s001 [file RA-010-C9RA09970K-s001.pdf]

Electronic Supplementary Material (ESI) for RSC Advances.
This journal is © The Royal Society of Chemistry 2019

Supporting Information for

Site-selective Doping effect, Phase Separation, and Structure Evolution in 1:1:1 Triple-Cation B-site Ordered Perovskites $Ca_{4-x}Sr_xGaNbO_8$

He Huang,[a] Pengfei Jiang,*[a,b] Wenliang Gao,[a] Rihong Cong,[a] Tao Yang*[a]

[a] College of Chemistry and Chemical Engineering, Chongqing University, Chongqing, 401331, P. R. China

[b] College of Physics, Chongqing University, Chongqing, 401331, P. R. China

* Corresponding authors: pengfeijiang@cqu.edu.cn, taoyang@cqu.edu.cn.

Table S1. Atomic coordinates, isotropic thermal displacement and site occupancy factors for $Ca_{4-x}Sr_xGaNbO_8$ (x = 0-4) obtained from Rietveld refinements against XRD data.

x = 0	x	y	z	Occ.	$U_{iso.}$ (Å^2)
Ca1(A1)	0.0703(3)	0.2438(7)	0.0986(2)	1	0.0255(4)
Ca2(A2)	0.6056(3)	0.7767(7)	0.4308(2)	1	0.0255(4)
Ca3(A3)	0.7547(3)	0.2705(7)	0.1224(2)	1	0.0255(4)
Ca4 (B1)	0.7098(3)	0.2295(7)	0.8593(2)	1	0.0255(4)
Nb	0.9244(1)	0.7502(2)	0.6319(1)	1	0.0161(4)
Ga	0.6103(2)	0.7083(3)	0.6721(1)	1	0.0183(6)
O1	0.5660(7)	0.1794(17)	0.4321(6)	1	0.0242(9)
O2	0.0897(8)	0.8165(14)	0.1398(6)	1	0.0242(9)
O3	0.7314(7)	0.8107(15)	0.6228(6)	1	0.0242(9)
O4	0.3246(7)	0.1692(14)	0.1942(6)	1	0.0242(9)
O5	0.3853(8)	0.6178(13)	0.3199(7)	1	0.0242(9)
O6	0.0113(9)	0.0782(15)	0.7217(7)	1	0.0242(9)
O7	0.1708(9)	0.978(15)	0.9638(7)	1	0.0242(9)
O8	0.0850(9)	0.9742(15)	0.5061(7)	1	0.0242(9)
x = 0.25	x	y	z	Occ.	$U_{iso.}$ (Å^2)
Ca1/Sr1(A1)	0.7087(3)	0.2303(8)	0.8595(3)	0.838(6)/0.162(6)	0.028(7)
Ca2/Sr2(A2)	0.7555(3)	0.2494(9)	0.1239(3)	0.922(5)/0.078(5)	0.028(4)
Ca3/Sr3(A3)	0.6034(3)	0.7777(9)	0.4314(2)	0.983(5)/0.017(5)	0.035(3)

Ca4/Sr4(B1)	0.0735(2)	0.2464(7)	0.1024(2)	0.972(7)/0.028(7)	0.017(5)
Nb	0.9232(2)	0.7511(4)	0.6310(1)	1	0.020(1)
Ga	0.6106(2)	0.7094(4)	0.6728(2)	1	0.019(1）
O1	0.5665(8)	0.1818(20)	0.4304(7)	1	0.029(2)
O2	0.0883(9)	0.8188(17)	0.1334(7)	1	0.029(2)
O3	0.7340(8)	0.8064(20)	0.6312(6)	1	0.029(2)
O4	0.3218(8)	0.1665(17)	0.1892(7)	1	0.029(2)
O5	0.3850(10)	0.6185(15)	0.3238(8)	1	0.029(2)
O6	0.0075(10)	0.0663(19)	0.7247(8)	1	0.029(2)
O7	0.1660(10)	0.9816(18)	0.9644(8)	1	0.029(2)
O8	0.8528(10)	0.9746(18)	0.5074(8)	1	0.029(2)

$x = 1.0$	x	y	z	Occ.	$U_{iso.}$ (Å^2)
Ca1/Sr1(A1)	0.1409(1)	0.2628(4)	0.20983(6)	0.245(5)/0.754(5)	0.0174(7)
Ca2/Sr2(A2)	0.7261(3)	0.2833(6)	0.0473(1)	0.850(4)/0.150(4)	0.039(1)
Ca3/Sr3(A3)	0.4995(3)	0.7500(5)	0.12929(9)	0.862(4)/0.138(4)	0.0229(1)
Ca4/Sr4(B1)	0.9763(3)	0.7382(6)	0.1037(1)	0.959(4)/0.041(4)	0.026(1)
Nb	0.6626(2)	0.2496(3)	0.2096(1)	1	0.0178(6)
Ga	0.2587(2)	0.2096(4)	0.0536(1)	1	0.0182(7)
O1	0.4754(13)	0.4571(16)	0.2587(4)	1	0.0178(6)
O2	0.7932(11)	0.5142(14)	0.1799(4)	1	0.0178(6)
O3	0.8352(9)	0.2306(19)	0.2921(3)	1	0.0178(6)
O4	0.7943(11)	0.0423(14)	0.1548(4)	1	0.0178(6)

O5	0.4608(9)	0.2994(15)	0.1214(4)	1	0.0178(6)
O6	0.0533(10)	0.3521(13)	0.0823(3)	1	0.0178(6)
O7	0.3235(9)	0.3112(14)	0.9681(4)	1	0.0178(6)
O8	0.2424(11)	0.8909(12)	0.0534(4)	1	0.0178(6)
x = 1.5	x	y	z	Occ.	$U_{iso.}$ (Å^2)
Ca1/Sr1(A1)	0.1424(2)	0.2618(5)	0.2101(1)	0.074(6)/0.926(6)	0.0213(9)
Ca2/Sr2(A2)	0.7255(3)	0.2805(7)	0.0475(1)	0.783(6)/0.217(6)	0.024(1)
Ca3/Sr3(A3)	0.7455(7)	0.7455(7)	0.1308(1)	0.623(5)/0.377(5)	0.021(1)
Ca4/Sr4(B1)	0.9761(4)	0.7380(9)	0.1039(1)	0.965(5)/0.035(5)	0.021(1)
Nb	0.6623(2)	0.2489(5)	0.2097(1)	1	0.0154(7)
Ga	0.2579(3)	0.2084(5)	0.0531(1)	1	0.0189(9)
O1	0.4779(16)	0.4619(21)	0.2579(6)	1	0.026(1)
O2	0.7947(14)	0.5147(19)	0.1781(6)	1	0.026(1)
O3	0.8313(11)	0.2533(26)	0.2923(4)	1	0.026(1)
O4	0.7917(14)	0.0392(19)	0.1584(5)	1	0.026(1)
O5	0.4607(12)	0.2933(21)	0.1221(4)	1	0.026(1)
O6	0.0516(12)	0.3593(16)	0.0838(5)	1	0.026(1)
O7	0.3230(12)	0.3145(17)	0.9689(5)	1	0.026(1)
O8	0.2306(13)	0.8921(16)	0.0547(5)	1	0.026(1)
x = 2.0	x	y	z	Occ.	$U_{iso.}$ (Å^2)
Ca1/Sr1(A1)	0.1428(2)	0.2621(6)	0.2101(1)	0.082(7)/0.918(7)	0.0216(9)
Ca2/Sr2(A2)	0.7278(3)	0.2797(7)	0.0471(1)	0.522(6)/0.478(6)	0.037(1)

Ca3/Sr3(A3)	0.4901(3)	0.7470(7)	0.1311(1)	0.371(6)/0.629(6)	0.024(1)
Ca4/Sr4(B1)	0.9739(4)	0.7391(11)	0.1042(2)	0.930(6)/0.070(6)	0.033(1)
Nb	0.6626(2)	0.2099(5)	0.2096(1)	1	0.0212(9)
Ga	0.2571(3)	0.2494(6)	0.0533(1)	1	0.022(1)
O1	0.4735(19)	0.4664(25)	0.2546(7)	1	0.032(1)
O2	0.7922(16)	0.5154(20)	0.1778(6)	1	0.032(1)
O3	0.8299(12)	0.2510(28)	0.2914(5)	1	0.032(1)
O4	0.7953(16)	0.0368(21)	0.1597(6)	1	0.032(1)
O5	0.4576(13)	0.2810(25)	0.1202(4)	1	0.032(1)
O6	0.0595(15)	0.3631(18)	0.0839(5)	1	0.032(1)
O7	0.3255(13)	0.3096(20)	0.9710(5)	1	0.032(1)
O8	0.2257(15)	0.9000(16)	0.0537(5)	1	0.032(1)
x = 2.5	x	y	z	Occ.	$U_{iso.}$ (Å^2)
Ca1/Sr1(A1)	0.1435(2)	0.26725	0.2102(1)	0.004(9)/0.996(9)	0.0147(9)
Ca2/Sr2(A2)	0.7271(3)	0.2807(6)	0.0469(1)	0.332(7)/0.668(7)	0.017(1)
Ca3/Sr3(A3)	0.4868(3)	0.7521(5)	0.1329(1)	0.143(8)/0.857(8)	0.0155(9)
Ca4/Sr4(B1)	0.9725(5)	0.7466(10)	0.1049(2)	0.926(6)/0.074(6)	0.013(1)
Nb	0.6633(2)	0.2550(5)	0.2098(1)	1	0.0107(8)
Ga	0.2571(3)	0.2162(6)	0.0536(1)	1	0.014(1)
O1	0.4750(21)	0.4732(27)	0.2492(7)	1	0.016(1)
O2	0.7905(17)	0.5224(20)	0.1780(6)	1	0.016(1)
O3	0.8284(12)	0.2332(27)	0.2939(5)	1	0.016(1)

O4	0.8033(18)	0.0424(21)	0.1635(6)	1	0.016(1)
O5	0.4600(13)	0.2653(27)	0.1224(4)	1	0.016(1)
O6	0.0633(16)	0.3667(19)	0.0863(5)	1	0.016(1)
O7	0.3217(14)	0.3025(21)	0.9694(5)	1	0.016(1)
O8	0.2143(16)	0.8996(17)	0.0555(5)	1	0.016(1)
x = 3.0	*x*	*y*	*z*	Occ.	$U_{iso.}$ (Å^2)
Ca1/Sr1(A1)	0.1434(3)	0.2693(6)	0.2099(1)	1	0.019(1)
Ca2/Sr2(A2)	0.7226(3)	0.2795(6)	0.0466(1)	0.088(7)/0.912(7)	0.020(1)
Ca3/Sr3(A3)	0.4846(3)	0.7539(6)	0.1335(1)	0.019(11)/0.981(8)	0.017(1)
Ca4/Sr4(B1)	0.9714(5)	0.7535(10)	0.1045(2)	0.809(8)/0.191(8)	0.019(1)
Nb	0.6636(3)	0.2186(7)	0.0542(1)	1	0.016(1)
Ga	0.2583(4)	0.2577(5)	0.2100(1)	1	0.017(1)
O1	0.4806(28)	0.4841(34)	0.2495(9)	1	0.020(1)
O2	0.7916(21)	0.5284(23)	0.1785(7)	1	0.020(1)
O3	0.8298(15)	0.2321(30)	0.2935(6)	1	0.020(1)
O4	0.7987(22)	0.0413(26)	0.1651(7)	1	0.020(1)
O5	0.4548(16)	0.2735(30)	0.1245(5)	1	0.020(1)
O6	0.0616(18)	0.3625(21)	0.0844(6)	1	0.020(1)
O7	0.3230(17)	0.2946(26)	0.9709(6)	1	0.020(1)
O8	0.2143(19)	0.9015(20)	0.0570(6)	1	0.020(1)
x = 3.5	*x*	*y*	*z*	Occ.	$U_{iso.}$ (Å^2)
Ca1/Sr1(A1)	0.1433(2)	0.2719(5)	0.2103(1)	1	0.0191(9)

Ca2/Sr2(A2)	0.7151(2)	0.2788(5)	0.0473(1)	0.027(6)/0.973(6)	0.021(1)
Ca3/Sr3(A3)	0.4896(2)	0.7529(5)	0.1353(1)	0.003(6)/0.997(6)	0.016(1)
Ca4/Sr4(B1)	0.9720(3)	0.7518(6)	0.1013(1)	0.421(6)/0.579(6)	0.021(1)
Nb	0.6626(2)	0.2582(4)	0.2103(1)	1	0.0161(9)
Ga	0.2585(3)	0.2143(5)	0.0553(1)	1	0.012(1)
O1	0.4642(18)	0.4858(26)	0.2505(7)	1	0.015(1)
O2	0.7953(16)	0.5165(20)	0.1797(6)	1	0.015(1)
O3	0.8180(11)	0.2458(28)	0.2942(4)	1	0.015(1)
O4	0.7885(17)	0.0323(20)	0.1622(5)	1	0.015(1)
O5	0.4578(13)	0.2924(21)	0.1268(4)	1	0.015(1)
O6	0.0617(14)	0.3587(17)	0.0842(5)	1	0.015(1)
O7	0.3359(14)	0.2945(21)	0.9746(5)	1	0.015(1)
O8	0.2326(15)	0.8973(17)	0.0582(5)	1	0.015(1)
x = 4.0	x	y	z	Occ.	$U_{iso.}$ (Å^2)
Ca1/Sr1(A1)	0.1426(2)	0.2757(5)	0.2106(1)	1	0.0228(9)
Ca2/Sr2(A2)	0.7062(2)	0.2757(5)	0.0475(1)	1	0.0228(9)
Ca3/Sr3(A3)	0.4943(2)	0.7501(6)	0.1365(1)	1	0.0228(9)
Ca4/Sr4(B1)	0.9739(2)	0.7509(6)	0.0989(1)	1	0.0228(9)
Nb	0.6630(2)	0.2599(5)	0.2111(1)	1	0.001(1)
Ga	0.2601(3)	0.2136(6)	0.0556(1)	1	0.018(1)
O1	0.4736(22)	0.5083(32)	0.2505(8)	1	0.021(1)
O2	0.7816(19)	0.5220(23)	0.1787(7)	1	0.021(1)

O3	0.8200(13)	0.2280(30)	0.2935(6)	1	0.021(1)
O4	0.7765(19)	0.0317(23)	0.1633(6)	1	0.021(1)
O5	0.4593(13)	0.2931(24)	0.1237(5)	1	0.021(1)
O6	0.0616(16)	0.35412(20)	0.0818(5)	1	0.021(1)
O7	0.3482(15)	0.3052(21)	0.9774(6)	1	0.021(1)
O8	0.2406(15)	0.9001(18)	0.0566(6)	1	0.021(1)

Table S2. Selected interatomic distances (Å) for $Ca_{4-x}Sr_xGaNbO_8$ (x = 0, 0.25, 1, 1.5, 2, 2.5, 3, 3.5, and 4) obtained from Rietveld refinements against XRD data.

**Ca_4GaNbO_8**					
Bond	Length (Å)	Bond	Length (Å)	Bond	Length (Å)
B1-O2	2.251(10)	Ga-O4	1.763(8)	Nb-O2	1.832(10)
B1-O4	2.319(9)	Ga-O5	1.825(7)	Nb-O7	1.936(8)
B1-O5	2.326(9)	Ga-O1	1.845(7)	Nb-O8	1.963(9)
B1-O1	2.370(11)	Ga-O3	1.906(10)	Nb-O6	2.034(9)
B1-O7	2.420(9)			Nb-O3	2.122(9)
B1-O8	2.465(8)			Nb-O6	2.149(8)

**$Ca_{3.75}Sr_{0.25}GaNbO_8$**					
Bond	Length (Å)	Bond	Length (Å)	Bond	Length (Å)
B1-O2	2.245(12)	Ga-O4	1.814(10)	Nb-O2	1.875(11)
B1-O4	2.300(10)	Ga-O5	1.839(9)	Nb-O7	1.917(9)
B1-O1	2.334(12)	Ga-O1	1.843(8)	Nb-O8	1.944(10)
B1-O5	2.375(10)	Ga-O3	1.845(11)	Nb-O6	2.037(10)
B1-O7	2.424(10)			Nb-O6	2.109(10)
B1-O8	2.480(10)			Nb-O3	2.148(10)

**$Ca_{3.35}Sr_{0.65}GaNbO_8$**					
Bond	Length (Å)	Bond	Length (Å)	Bond	Length (Å)
B1-O6	2.251(9)	Ga-O8	1.790(8)	Nb-O2	1.879(10)
B1-O3	2.257(8)	Ga-O6	1.851(9)	Nb-O3	1.883(7)
B1-O8	2.311(9)	Ga-O7	1.853(9)	Nb-O4	1.896(10)
B1-O7	2.380(8)	Ga-O5	1.888(8)	Nb-O1	2.039(10)
B1-O4	2.407(10)			Nb-O1	2.075(10)
B1-O2	2.479(10)			Nb-O5	2.088(7)

**$Ca_{3.25}Sr_{0.75}GaNbO_8$**					
Bond	Length (Å)	Bond	Length (Å)	Bond	Length (Å)
B1-O6	2.253(9)	Ga-O8	1.787(8)	Nb-O2	1.879(9)
B1-O3	2.268(8)	Ga-O6	1.810(9)	Nb-O3	1.887(8)
B1-O8	2.339(9)	Ga-O7	1.844(9)	Nb-O4	1.896(9)
B1-O7	2.387(8)	Ga-O5	1.856(8)	Nb-O1	2.029(11)
B1-O4	2.422(11)			Nb-O1	2.086(11)

B1-O2	2.456(10)			Nb-O5	2.103(7)
**$Ca_3SrGaNbO_8$**					
Bond	Length (Å)	Bond	Length (Å)	Bond	Length (Å)
B1-O3	2.280(6)	Ga-O6	1.795(7)	Nb-O2	1.880(8)
B1-O6	2.301(7)	Ga-O8	1.806(7)	Nb-O4	1.893(8)
B1-O8	2.368(8)	Ga-O7	1.858(7)	Nb-O3	1.894(6)
B1-O7	2.400(7)	Ga-O5	1.888(6)	Nb-O1	2.048(9)
B1-O4	2.420(8)			Nb-O1	2.072(9)
B1O2	2.420(9)			Nb-O5	2.108(6)
**$Ca_{2.5}Sr_{1.5}GaNbO_8$**					
Bond	Length (Å)	Bond	Length (Å)	Bond	Length (Å)
B1-O6	2.263(10)	Ga-O8	1.810(9)	Nb-O4	1.857(10)
B1-O8	2.296(10)	Ga-O6	1.842(10)	Nb-O3	1.890(8)
B1-O3	2.298(7)	Ga-O7	1.852(10)	Nb-O2	1.913(11)
B1-O2	2.397(11)	Ga-O5	1.905(8)	Nb-O1	2.044(12)
B1-O7	2.416(9)			Nb-O1	2.075(12)
B1-O4	2.461(11)			Nb-O5	2.104(7)
**$Ca_2Sr_2GaNbO_8$**					
Bond	Length (Å)	Bond	Length (Å)	Bond	Length (Å)
B1-O6	2.279(12)	Ga-O8	1.786(10)	Nb-O4	1.874(12)
B1-O8	2.317(11)	Ga-O6	1.803(11)	Nb-O3	1.877(9)
B1-O3	2.332(9)	Ga-O7	1.824(10)	Nb-O2	1.914(12)
B1-O2	2.402(12)	Ga-O5	1.858(9)	Nb-O1	2.045(14)
B1-O4	2.444(12)			Nb-O1	2.087(14)
B1-O7	2.444(10)			Nb-O5	2.144(8)
**$Ca_{1.5}Sr_{2.5}GaNbO_8$**					
Bond	Length (Å)	Bond	Length (Å)	Bond	Length (Å)
B1-O8	2.238(12)	Ga-O6	1.798(12)	Nb-O4	1.872(13)
B1-O3	2.299(9)	Ga-O7	1.837(11)	Nb-O3	1.914(9)
B1-O6	2.315(12)	Ga-O8	1.845(10)	Nb-O2	1.920(12)
B1-O2	2.408(12)	Ga-O5	1.873(9)	Nb-O1	2.043(16)
B1-O7	2.411(10)			Nb-O1	2.099(15)
B1O4	2.441(13)			Nb-O5	2.106(8)
**$CaSr_3GaNbO_8$**					
Bond	Length (Å)	Bond	Length (Å)	Bond	Length (Å)

B1-O8	2.225(14)	Ga-O6	1.784(13)	Nb-O4	1.861(15)
B1-O3	2.322(11)	Ga-O7	1.818(13)	Nb-O3	1.917(11)
B1-O6	2.390(14)	Ga-O8	1.857(12)	Nb-O2	1.944(14)
B1-O7	2.426(12)	Ga-O5	1.872(11)	Nb-O1	2.059(20)
B1-O2	2.430(15)			Nb-O1	2.091(20)
B1-O4	2.458(16)			Nb-O5	2.109(10)

$Ca_{0.5}Sr_{3.5}GaNbO_8$

Bond	Length (Å)	Bond	Length (Å)	Bond	Length (Å)
B1-O8	2.306(11)	Ga-O6	1.791(10)	Nb-O3	1.879(8)
B1-O6	2.397(10)	Ga-O7	1.824(11)	Nb-O2	1.913(12)
B1-O3	2.404(8)	Ga-O8	1.844(10)	Nb-O4	1.914(12)
B1-O4	2.495(12)	Ga-O5	1.934(9)	Nb-O1	2.019(14)
B1-O7	2.518(10)			Nb-O5	2.083(8)
B1-O2	2.525(12)			Nb-O1	2.157(14)

Sr_4GaNbO_8

Bond	Length (Å)	Bond	Length (Å)	Bond	Length (Å)
B1-O8	2.367(11)	Ga-O6	1.786(12)	Nb-O3	1.878(10)
B1-O6	2.420(11)	Ga-O8	1.822(11)	Nb-O4	1.882(13)
B1-O3	2.448(10)	Ga-O7	1.844(12)	Nb-O2	1.899(14)
B1-O2	2.613(14)	Ga-O5	1.899(9)	Nb-O1	1.972(18)
B1-O4	2.613(13)			Nb-O5	2.135(9)
B1-O7	2.622(10)			Nb-O1	2.202(18)

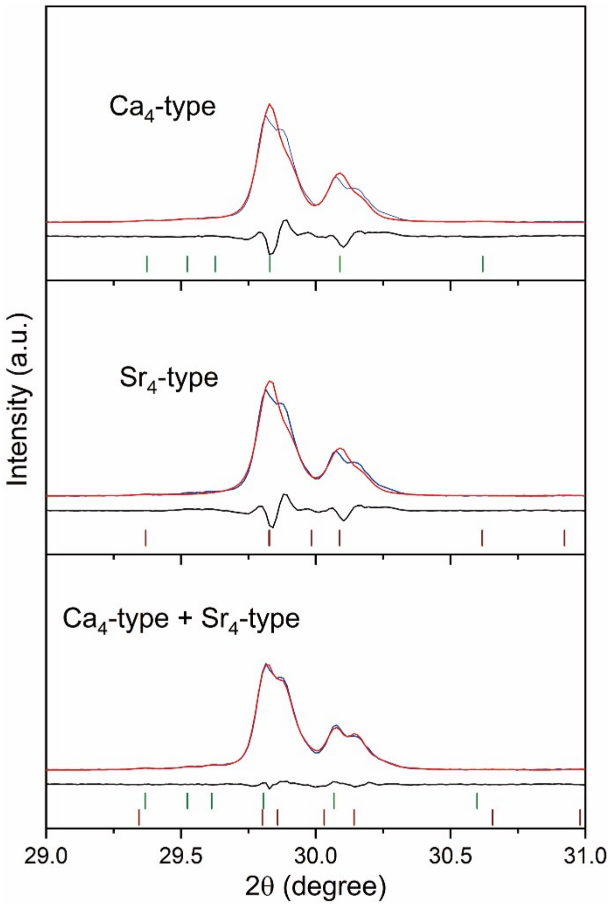

Figure S1. Le-bail fitting patterns for patterns for $Ca_{3.5}Sr_{0.5}GaNbO_8$ with different lattice parameters.

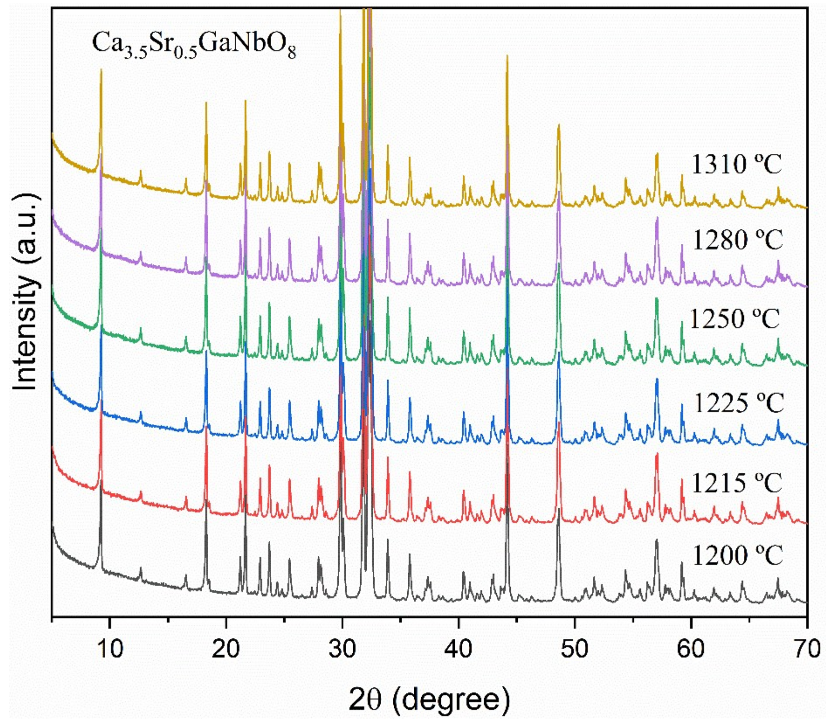

Figure S2. XRD patterns for $Ca_{3.5}Sr_{0.5}GaNbO_8$ after being heated at different temperatures.

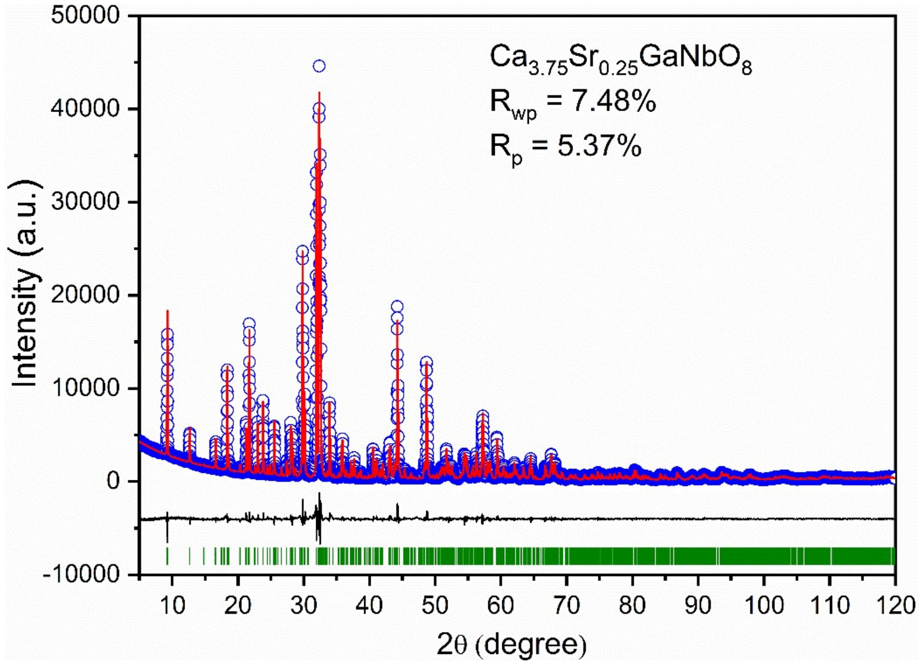

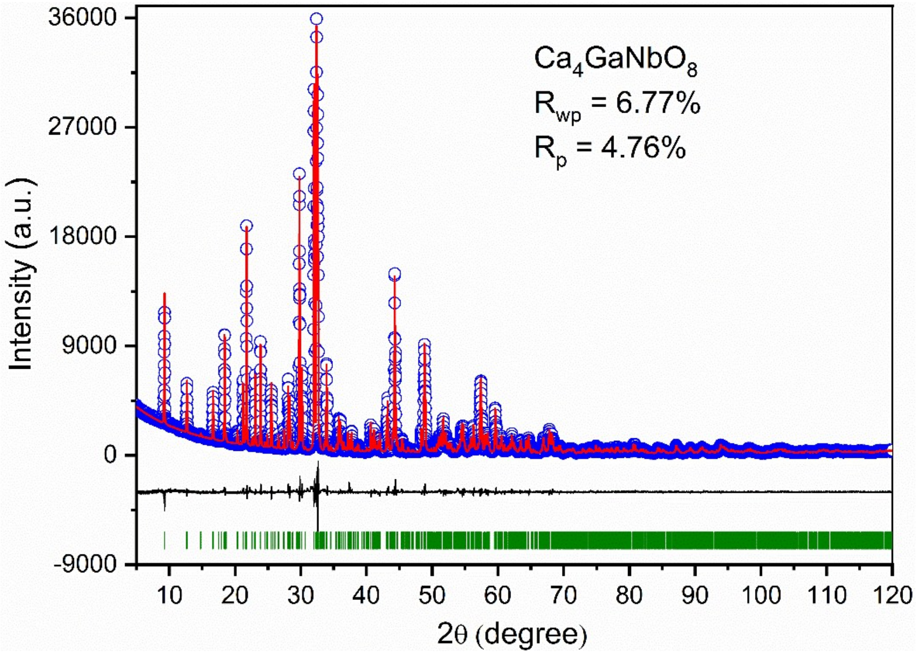

Ca_4GaNbO_8
R_{wp} = 6.77%
R_p = 4.76%
Intensity (a.u.)
2θ (degree)
36000
27000
18000
9000
0
-9000
10
20
30
40
50
60
70
80
90
100
110
120

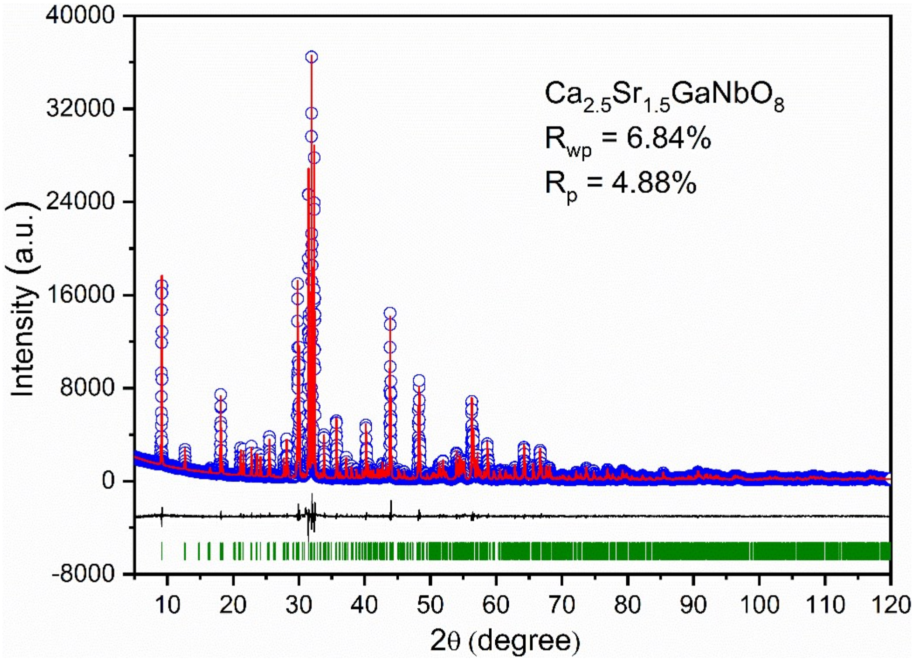

$Ca_{2.5}Sr_{1.5}GaNbO_8$
R_{wp} = 6.84%
R_p = 4.88%
Intensity (a.u.)
2θ (degree)
40000
32000
24000
16000
8000
0
-8000
10
20
30
40
50
60
70
80
90
100
110
120

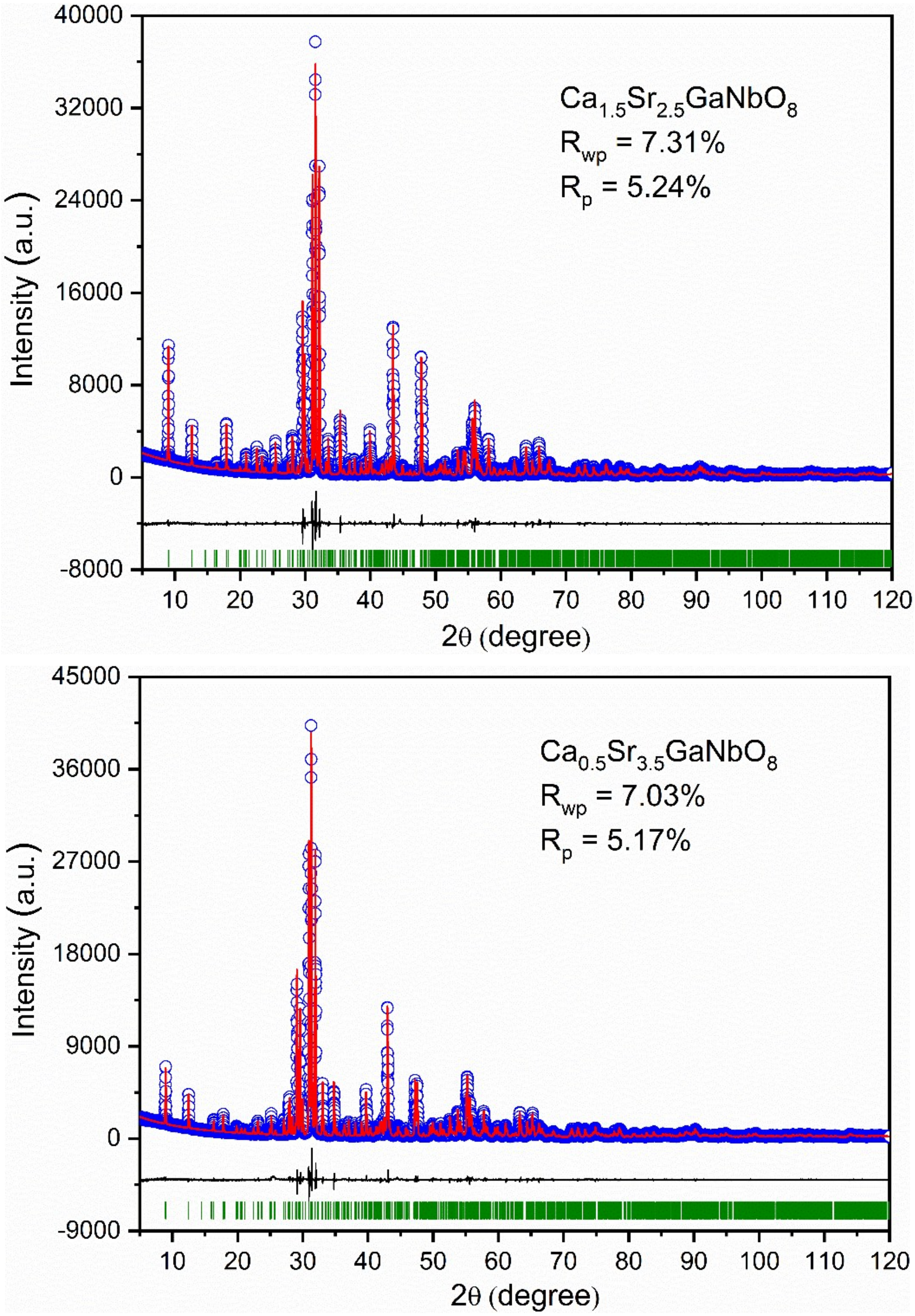

Figure S3. Rietveld refinement plot of XRD data for $Ca_{4-x}Sr_xGaNbO_8$ (x = 0, 1.5, 2.5, and 3.5). The blue ○ and red solid line and the black solid line represent the observed, calculated patterns, and the difference of these two, respectively. The reflection positions are shown as dark green bars at the bottom of the patterns. The refined agreement factors were also given in the patterns.

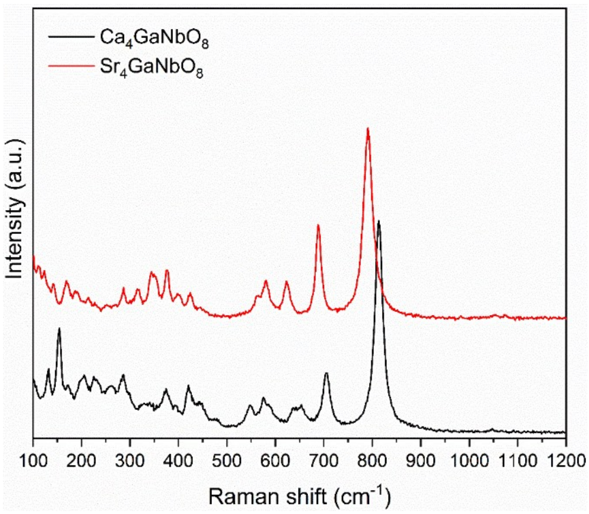

Figure S4. Raman spectra of Ca_4GaNbO_8 and Sr_4GaNbO_8.

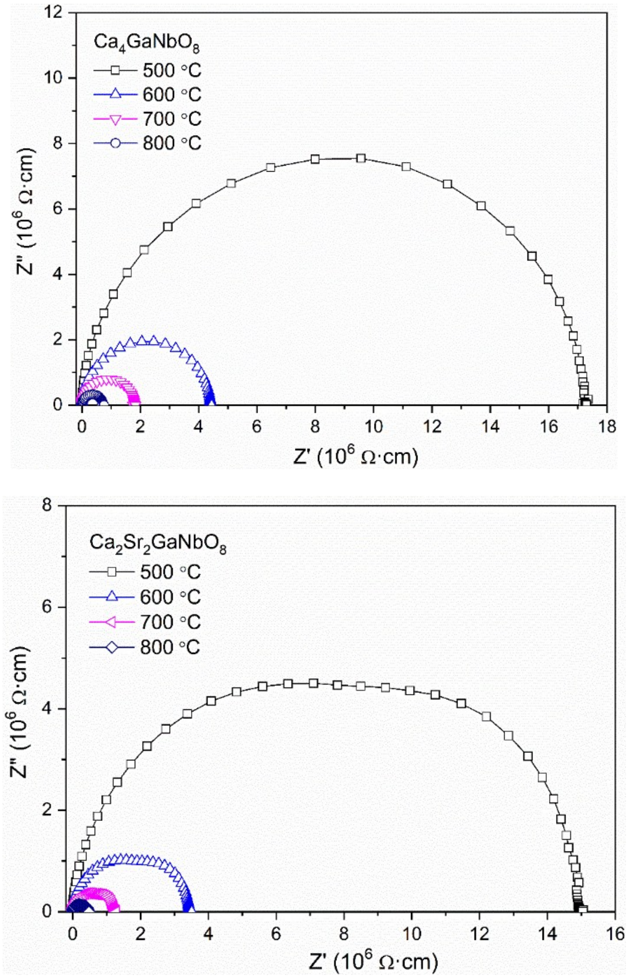

Figure S5. Complex impedance plots for Ca_4GaNbO_8 and $Ca_2Sr_2GaNbO_8$ at 500,600, 700 and 800 °C.
